# Damage Control Surgery for Hepatocellular Cancer Rupture in an Elderly Patient: Survival and Quality of Life

**DOI:** 10.1155/2015/536029

**Published:** 2015-10-04

**Authors:** Konstantinos Bouliaris, Grigorios Christodoulidis, Dimitrios Symeonidis, Alexandros Diamantis, Konstantinos Tepetes

**Affiliations:** Surgical Department, University Hospital of Larissa, Mezurlo, 4110 Larissa, Greece

## Abstract

Spontaneous rupture of hepatocellular carcinoma (HCC) is a rare emergency condition with high mortality rate. Successful management depends on patients' hemodynamic condition upon presentation and comorbidities, correct diagnosis, HCC status, liver function, and future liver remnant, as well as available sources. There is still a debate in the literature concerning the best approach in this devastating complication. Nevertheless, the primary goal should be a definitive bleeding arrest. In most cases, patients with spontaneous rupture of HCC present with hemodynamic instability, due to hemoperitoneum, necessitating an emergency treatment modality. In such cases, transcatheter arterial embolization (TAE) should be the treatment of choice. Emergency liver resection is an option when TAE fails or in cases with preserved liver function and limited tumors. Otherwise, damage control strategies, as in liver trauma, are a reasonable alternative. We report a case of an elderly patient with hemoperitoneum and hypovolemic shock from spontaneous rupture of undiagnosed HCC, who was treated successfully by emergency surgery and damage control approach.

## 1. Introduction

Hepatocellular carcinoma (HCC) is an aggressive tumor that often occurs in the setting of chronic liver disease and cirrhosis and it is typically diagnosed late in its course. It is the sixth most common malignancy in the world and the third commonest cause of death from cancer [1]. One of the most life-threatening conditions associated with HCC is spontaneous rupture which occurs in 3–26% of all HCC cases [2, 3]. Thirty-day mortality can be very high ranging from 32 to 75% [4]. This poor outcome can be attributed to incorrect diagnosis, concomitant impaired liver function, advanced HCC status, inappropriate treatment, and patient's general condition and comorbidities upon presentation. In most cases, patients with spontaneous rupture of HCC present with hemodynamic instability, due to hemoperitoneum, necessitating an emergency treatment modality. Therapeutic options include transcatheter arterial embolization (TAE), surgical ligation of the hepatic artery, perihepatic packing, oversewing of the bleeding surface, and hepatectomy. Efforts for hemostasis in such patients should be directed by the available sources and the hemodynamic status. Thus, when TAE is not available, surgery with or without hepatectomy should be the first choice. Herein, we present a case of a successful two-stage surgical treatment of a ruptured HCC in an elderly patient who presented with hypovolemic shock.

## 2. Case Presentation

**Figure 1:**
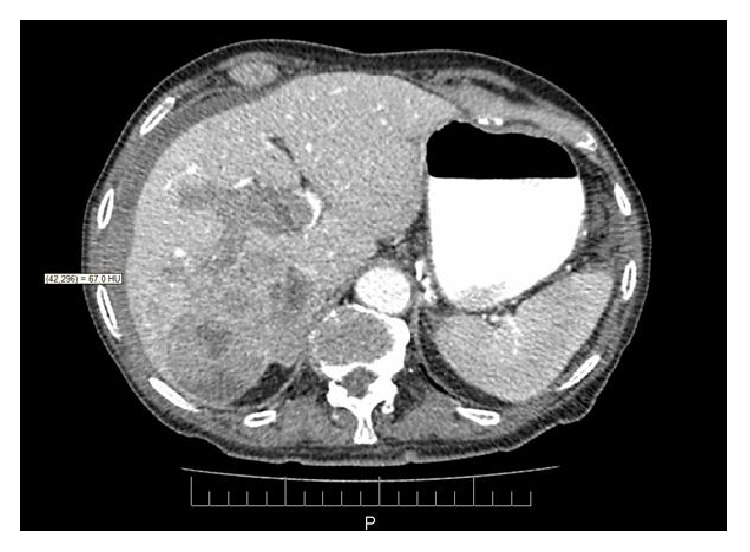
Abdomen CT showing a 7.5 cm mass occupying the right lobe of the liver, thrombosis of the right portal vein, and hemoperitoneum (HU = 67).

A 87-year-old male patient was transferred to the emergency department after an episode of sudden upper abdominal pain and vomiting. On arrival, the patient was pale, tachycardic with a heart rate of 103 beats per minute, and tachypnoeic with a blood pressure of 110/60 mmHg. Physical examination revealed guarding of the right upper quadrant with tenderness. Laboratory examination revealed a hemoglobin level of 10.2 g/dL, normal platelets count, prolonged INR = 1.41, normal liver enzymes, and slightly elevated *γ*GT = 70 U/I (normal values < 50). Past medical history included coronary artery disease with coronary artery bypass surgery and carotid artery stenting. However, electrocardiogram and cardiac enzymes were within normal values. Abdominal ultrasound showed a hepatic lesion with free intraperitoneal fluid. A contrast enhanced abdominal CT was ordered which demonstrated a heterogenous mass of 7.5 cm diameter occupying the right lobe of the liver, thrombosis of the right portal vein, and free quantity of blood in the peritoneal cavity (Figure 1). These findings indicated a spontaneous rupture of a possible HCC since there was no past history of HCC disease. During the examination, the patient became hemodynamically unstable, with loss of consciousness. He was intubated and transferred to the operating room for an emergency exploratory laparotomy since TAE was not feasible at that time. During surgery, there was a notable amount of fresh and clotted blood in the abdomen and a large hepatic ruptured mass was detected, located in the right hepatic lobe. Although a right hepatectomy was technically feasible, this was not performed due to critical patient's situation. Under these circumstances, it was decided to perform damage control surgery with enucleation of the tumor, ligation of the hepatic artery, and perihepatic packing. Patient's condition did not permit us to check intraoperatively the patency of the main portal vein, but the CT had shown that the left portal vein was patent and there was also a collateral circulation due to cirrhosis. The haemorrhage was successfully controlled and the patient was transferred to the intensive care unit (ICU) for further supportive treatment. Forty-eight hours later, a second laparotomy was performed to remove the packing and apply RF ablation to the tumor's bed. After 4 days in the ICU, the patient was transferred to surgical ward and he was discharged on the 18th postoperative day. The histopathological examination showed HCC, while serological tests were positive for hepatitis B virus infection. One year after the operation, he is still alive, in good condition living at his village.

## 3. Discussion

Hepatocellular cancer is a hypervascular tumor with a high tendency for vascular invasion. However, the mechanism of spontaneous rupture is still not exactly known. Possible causes are rapid growth and necrosis, erosion of a vessel, increased intratumoral pressure caused from the occlusion of hepatic veins by tumor thrombi or invasion, and coagulopathy [5]. Spontaneous rupture is the third most common cause of death due to HCC, after neoplastic progression and liver failure, and it is more frequent in males [6]. An important condition for intraperitoneal bleeding is tumor's location with tumors at the edge of the liver being at greater risk compared with intraparenchymal HCC. Typical symptoms of spontaneous rupture are epigastric pain of sudden onset with or without clinical signs of shock and peritoneal irritation. Diagnosis of ruptured HCC can be difficult especially when there is no history of HCC, cirrhosis, or HBV infection and the patient is in hemodynamic instability [5, 6]. Abdominal CT scans are useful in demonstrating the presence of hemoperitoneum and liver tumor with the additional advantage of showing tumor characteristics, the amount of ascites, related vascular abnormalities, patency of portal vein, and the likelihood of metastasis [4]. The primary aim of management in such cases should be the restoration and support of intravascular volume and attempts for hemostasis and preservation of liver parenchyma as much as possible [5, 6]. It is known that bleeding and hemodynamic instability are factors which strongly influence the prognosis [7–9]. Thus, the therapeutic choice must take into account the hemodynamic conditions, functional status of the liver, and stage of the cancer [6]. TAE has been increasingly used for hemostasis in ruptured HCC especially when there is hepatic insufficiency or liver cirrhosis [6, 10–12]. Even if TAE is associated with rebleeding, liver abscess, and a mortality rate of around 30%, this technique remains the best method to achieve hemostasis without surgery with a success rate up to 99% in cases of ruptured HCC [11, 13–15]. Moreover, when feasible, super selective TAE has the advantage of preserving liver function and can be used either as a definitive treatment or as a bridge to liver resection. When TAE fails or is not available, surgical hemostasis is the only alternative. Surgical hemostasis can be achieved by various techniques, including perihepatic packing, suture plication of bleeding tumor, hepatic artery ligation, and liver resection. Although open surgical procedures achieve a high rate of hemostasis and better survival outcome than TAE [16], they are associated with a high in-hospital mortality rate especially following emergency liver resections due to the damage of the residual hepatic function, leading to postoperative liver failure [4]. Furthermore, the presence of hemorrhagic shock renders the liver function poorer than usual. Therefore, emergency one-stage liver resection is feasible in patients with adequate liver function and limited tumors [5]. When patient's condition is too critical for emergency liver resection, a damage control strategy should be selected, as in liver trauma cases. In our patient, a combination of enucleation of the tumor, perihepatic packing, and hepatic artery ligation (HAL) was performed due the hemodynamic instability and his comorbidities associated with his age. In ruptured HCC, HAL has a hemostatic success rate of 68% to 100% and can either be selective or at the level of the hepatic artery, but its use is limited due to its high in-hospital mortality rate of 50% to 77% [17–19]. Packing of a bleeding tumor achieves hemostasis by tamponade effect and it is effective especially for oozing tumors. The duration of pack should be 24 to 48 hours. Packing for longer time increases the rate of intra-abdominal infection and sepsis according to clinical evidence from perihepatic packing in liver trauma cases [20, 21]. Perihepatic packing is a reliable method in hemodynamically unstable patients who require a fast damage control laparotomy providing early resuscitation and stabilization.

In summary, the management of spontaneous rupture of HCC is always a troublesome situation for emergency physicians and surgeons. The treatment of this devastating emergency should be closely scrutinized, with stabilization of vital signs and maintenance of hepatic perfusion as soon as possible. Nevertheless, the primary goal should be a definitive bleeding arrest. Although there is no consensus on the most effective treatment, the therapeutic choice must take into account the hemodynamic conditions, functional status of the liver, and stage of the cancer. In general, TAE should be the method of choice as it is less invasive and very efficacious technique and can be done with regional anesthesia. However, for patients with stable vital signs, noncirrhotic liver, and limited tumors, as shown in the abdominal CT scan, immediate hepatectomy can also be considered [4]. For unstable patients with poor liver function or questionable posthepatectomy functional residual volumes, TAE is very effective in achieving immediate hemostasis [4, 6]. Whether TAE is to be followed by staged hepatectomy depends on the recovery of liver function and thorough investigation of the tumor characteristics [4]. In cases of unstable patients and TAE failure, surgical hemostasis should be performed. Liver resection maybe attempted in cases with small tumors, adequate FLR (future liver remnant), and well preserved liver function but with a high rate of mortality. Otherwise, damage control strategies, as in liver trauma, are a reasonable alternative.
